# 

**Published:** 1898-10-22

**Authors:** 


					TEE HOSPITAL. " The standard of highest purity ."?Lancet. 0| |^^^1898,
CADo^.RY's
ABSOLUTELY PURE. ?=> , NO DRUGS OR ALKALIES USED.
]RW!CH HOSPUAX
No. 630. Vol. XXV. ESfiSSSa SATURDAY,
Oot. 22nd, 1898.
[Sub.^ptionTerms,] pRICE TWOPENCE.

				

